# Supplementary material for: Long-term consequences of benzodiazepine-induced neurological dysfunction: A survey
Source: PLoS One. 2023 Jun 29;18(6):e0285584. doi: 10.1371/journal.pone.0285584 (PMC10309976; doi:10.1371/journal.pone.0285584)
Supplement: S1 Appendix — (DOCX) [file pone.0285584.s001.docx]

**Appendix 1**

Survey Flow

Question 1: Welcome

The intent of this survey is to document experiences people have had or currently have due to benzodiazepine use or withdrawal, possibly taken with other medications, including experiences with extended symptoms over years. This is the first survey to attempt to document the frequency of certain experiences, so we very much want to hear your personal experience. We hope to hear from hundreds of people if possible.

All responses are completely anonymous. By taking this survey, you are giving consent for use of your numerical data to assess the frequency of symptoms across many people. IP addresses are used in this study ONLY to ensure an IP address can complete the survey only once. When data collection is completed, IP addresses will be permanently deleted from the data (a serious promise).

This survey cannot list every possible symptom because there is a constellation of them; there is an open-ended text box at the end of the survey for anything more you want to tell us.

The questionnaire should take about 10 minutes – or complete part and return later.

Question 2: Which medication did or do you take? Choose all that apply.

|  | Xanax/alprazolam (1) |
| --- | --- |
|  | Valium/diazepam (2) |
|  | Klonopin/clonazepam (3) |
|  | Ativan/lorazepam (4) |
|  | Librium/cholorodiazepoxide (5) |
|  | Onfi/clobazam (6) |
|  | Restoril/temazepam/Normison (7) |
|  | Halcion/triazolam (8) |
|  | Prosom, Serax, Traxene, or Serepax (9) |
|  | Other that you are sure is a benzodiazepine (10) |
|  | Z-drug such as Ambien/zolpidem, Sonata/zaleplon (11) |
|  | Abilify/aripiprazole, Risperdal/risperidone, Zyprexa/olanzapine or other anti-psychotic (12) |
|  | Neurontin/gabapentin or Lyrica/pregabalin (13) |
|  | Antidepressants such as Prozac, Paxil, Celexa, Effexor, or other (15) |
|  | Have not taken any of these (14) |
|  | Have not taken a benzodiazepine (Z-class meds are not benzos) (16) |

Question 3: Did you take different medications concurrently (at the same time) or sequentially (different ones at different times)?

|  | Sequentially, different ones at different times (1) |
| --- | --- |
|  | Concurrently, two or more at the same times (2) |
|  | Only took a benzodiazepine (4) |

Question 4: For which condition/situation was the medication originally prescribed? Check all that apply.

|  | Situational anxiety (1) |
| --- | --- |
|  | PMS (2) |
|  | Insomnia, sleep (3) |
|  | Panic attacks (4) |
|  | GAD (5) |
|  | Restless legs (6) |
|  | Seizures (7) |
|  | Hallucinations or schizophrenia (8) |
|  | Muscle spasms or clenched muscles (9) |
|  | Pain or nerve spasms (14) |
|  | Part of cancer or other major illness/accident treatment assistance (10) |
|  | Depression (11) |
|  | Other (12) |
|  | Not prescribed (13) |

Question 5: Did you quit a benzodiazepine abruptly, or if you tapered, how long did your taper last? Please answer ONLY for benzodiazepines. If you are still tapering, click the last answer and go on to the next question.

|  | Still taking a full dose (1) |
| --- | --- |
|  | Attempted to taper, went back on the med due to symptoms (2) |
|  | Stopped abruptly, withdrew just fine without taper (9) |
|  | Did not taper, quit abruptly but with consequences (3) |
|  | Tapered over weeks or days (4) |
|  | Tapered 1-6 months (5) |
|  | Tapered 6-12 months (6) |
|  | Tapered between 1-2 years (7) |
|  | Tapered more than 2 years (8) |
|  | If you are still tapering, click this button and go to the next question (12) |

Question 6: If you are still tapering, how long has your taper lasted so far? If you have stopped tapering or are still taking a full dose, choose “does not apply to me”.

|  | Weeks or days (1) |
| --- | --- |
|  | 1-6 months (2) |
|  | 7-12 months (3) |
|  | Between 1-2 years (4) |
|  | 2 years or more (5) |
|  | Does not apply to me (6) |

Question 7: From this point, please **only answer questions for benzodiazepine use**, separate from other medications.

When was the last time you took a benzodiazepine dose?

|  | Today or recent days (1) |
| --- | --- |
|  | Weeks (2) |
|  | Months (3) |
|  | 1 Year or More (4) |

Question 8: Was your benzodiazepine med always taken ‘as prescribed’, in the dose recommended by a doctor?

|  | Definitely yes (1) |
| --- | --- |
|  | Mostly yes (2) |
|  | Definitely not (3) |

Question 9: In the left column are medication symptoms that may have occurred, including during a benzodiazepine ongoing dose or long after ceasing the prescription, and on the right, buttons indicating how long that symptom lasted for you. Please answer only for your benzodiazepine experience, although symptoms may be similar for other medications listed early.

For each potential symptom, click the button that best explains how long each symptom lasted, or “did not experience” if that is the case. That way, we know what you experienced, and for how long.

**For how long did a symptom last?**

|  | Did not experience this (1) | Days (2) | Weeks (3) | Months (4) | 1 or more years (5) |
| --- | --- | --- | --- | --- | --- |
| Whole or partial body seizures (1) |  |  |  |  |  |
| Muscle spasms in back or limbs (2) |  |  |  |  |  |
| Difficulty breathing or swallowing (3) |  |  |  |  |  |
| Muscle weakness (4) |  |  |  |  |  |
| Stabbing pain, burning, aching sensations, or joint pain (5) |  |  |  |  |  |
| Digestion, nausea, diarrhea, other stomach/gut issue (6) |  |  |  |  |  |
| Head pain, pressure (7) |  |  |  |  |  |
| Heart rhythm irregularities or high blood pressure (8) |  |  |  |  |  |

Question 10: Other symptoms may also be experienced. Click the button that best describes benzodiazepine withdrawal or other symptoms you may have experienced over time, and for how long it lasted.

**For how long did each symptom last?**

|  | Did not experience this (1) | Days (2) | Weeks (3) | Months (4) | 1 or more years (5) |
| --- | --- | --- | --- | --- | --- |
| Whole body trembling uncontrollably (1) |  |  |  |  |  |
| Balance problems (2) |  |  |  |  |  |
| Trembling or tingling in limbs, skin (3) |  |  |  |  |  |
| Sleep disturbances (4) |  |  |  |  |  |
| Difficulty focusing, distracted (5) |  |  |  |  |  |
| Low Energy (6) |  |  |  |  |  |
| Difficulty driving or walking (7) |  |  |  |  |  |
| Memory loss (8) |  |  |  |  |  |
| Hallucinations (9) |  |  |  |  |  |
| Akathisia, need to move or pace constantly (10) |  |  |  |  |  |

Question 11: Below are another set of symptoms that you may have experienced due to discontinuing or tapering a benzodiazepine. Click the button that best describes symptoms you may have experienced.

For how long did a symptom last?

|  | Did not experience this (1) | Days (2) | Weeks (3) | Months (4) | 1 or more years (5) |
| --- | --- | --- | --- | --- | --- |
| Nervousness, anxiety, fear (1) |  |  |  |  |  |
| Uncontrollable crying or anger (2) |  |  |  |  |  |
| No appetite, disinterest in food (3) |  |  |  |  |  |
| Symptoms triggered or worsened by foods, alcohol, or caffeine (4) |  |  |  |  |  |
| Sensitivity to light, noise, smell, triggering symptoms (5) |  |  |  |  |  |

Question 12: Where you warned that benzodiazepines should only be taken for short times, or that they are difficult to withdraw from?

|  | Yes, clearly warned (1) |
| --- | --- |
|  | Yes, but not sufficiently (2) |
|  | Can’t remember (3) |
|  | Probably not (4) |
|  | Definitely not (5) |

Question 13: It may be important to know how benzodiazepine discontinuation symptoms have affected your life. To what extent has your condition affected your work or personal life?

**How severely did this problem affect your…**

|  | Not at all (1) | Mild problem (2) | Moderate problem (3) | Severe problem (4) | Quite severe problem (5) | Enormous problem (6) |
| --- | --- | --- | --- | --- | --- | --- |
| Work life (1) |  |  |  |  |  |  |
| Fun, recreation, hobbies (2) |  |  |  |  |  |  |
| Ability to take care of home, others (3) |  |  |  |  |  |  |
| Ability to drive or walk (4) |  |  |  |  |  |  |
| Social interaction, friendships (5) |  |  |  |  |  |  |
| Relationships with spouse, family (6) |  |  |  |  |  |  |

Question 14: Specifically, have any of these been consequences of your benzodiazepine use or withdrawal? Choose all that apply, or none if that is the case.

|  | Lost a job, fired, became unable to work (1) |
| --- | --- |
|  | Loss of wages or lower wages in a reduced job capacity (2) |
|  | Lost a business, if a business owner (3) |
|  | Significantly affected marriage, other relationships (4) |
|  | Lost child custody (5) |
|  | Lost a home (6) |
|  | Experienced significant increase in medical costs (7) |
|  | Lost savings or retirement funds (8) |
|  | None of these apply (9) |
|  | Suicidal thoughts or attempted suicide (10) |
|  | Violent thoughts or actual violence against others (11) |

Question 15: Are any of the following symptoms true for you?

|  | Not a problem (1) | Yes, and occurred prior to benzo use (2) | Yes, occurred only after benzo use (3) |
| --- | --- | --- | --- |
| Food and/or seasonal allergies (1) |  |  |  |
| Highly sensitive airways (2) |  |  |  |
| Sensitive to food additives or chemicals (3) |  |  |  |
| Allergic/sensitive to MSG or soy (4) |  |  |  |
| Diagnosed autoimmune disorder (5) |  |  |  |
| Falls or fractures (6) |  |  |  |

Question 16: Would you please give your gender? It may be important to know.

|  | Male (1) |
| --- | --- |
|  | Female (2) |
|  | Other gender identity (3) |
|  | Prefer not to say (4) |

Question 17: It could be helpful to track symptoms by age. Your age group currently is:

|  | Under 20 (1) |
| --- | --- |
|  | 20-30 (2) |
|  | Over 30, under 50 (3) |
|  | Over 50, under 60 (4) |
|  | 60 or over (5) |

Question 18: What country do you live in?

|  | U.S. (1) |
| --- | --- |
|  | U.K. (2) |
|  | Canada (3) |
|  | Australia (4) |
|  | Japan (5) |
|  | Germany (6) |
|  | France (7) |
|  | Denmark (8) |
|  | Ireland (9) |
|  | Other ______________ (10) |

Question 19: Where did you learn about this survey?

|  | A benzodiazepine support group (1) |
| --- | --- |
|  | Word of mouth, a friend (2) |
|  | Internet search (3) |

Question 20: If there is anything you’d particularly like to tell us, you can do so in this open-ended text box:

__________________________________________________________________
