# Supplementary material for: Long-term consequences of benzodiazepine-induced neurological dysfunction: A survey
Source: PLoS One. 2023 Jun 29;18(6):e0285584. doi: 10.1371/journal.pone.0285584 (PMC10309976; doi:10.1371/journal.pone.0285584)
Supplement: S2 Appendix — (DOCX) [file pone.0285584.s002.docx]

**Appendix II**

**The Benzodiazepine Nosology Workgroup**

Twenty-three experts with academic, clinical, and/or lived experience taking benzodiazepines formed the Benzodiazepine Nosology Workgroup to derive a clinically serviceable, scientifically accurate, and non-stigmatizing name for this condition. In a series of meetings and using the Delphi method, a variety of terms were evaluated. As a result of this process, benzodiazepine-induced neurological dysfunction and its acronym BIND have been proposed to the benzodiazepine community and now, in this article, to the wider medical community, neuroscientists, and all of those responsible for medical nomenclature to describe the symptoms induced by the use of benzodiazepines that manifest as neurological dysfunction. BIND may begin while taking benzodiazepines and can persist for weeks, months, or even years after discontinuation. This new term defines a distinct and often overlooked proportion of benzodiazepine users and endorses the validity of their experiences with a constellation of enduring, adverse, and even disabling symptoms.

The term BIND may provide impetus for much-needed research into the underlying molecular mechanisms of both acute and enduring symptoms to facilitate diagnosis, treatment, and management strategies. Clear terminology will aid in quantification of this condition and allow clinicians and medical researchers to better understand the scope, effect, and pathophysiology associated with prolonged benzodiazepine exposure. An apt name for this condition may also serve to raise awareness among prescribers about the risks associated with benzodiazepine prescribing, particularly when these medications are prescribed off-label or used for extended courses of treatment.

The members of this group, in alphabetical order, are: Sumit Agarwal, MD, Harvard Medical School and Brigham and Women’s Hospital, Boston, Massachusetts USA; Richard Bailey, BSc (Hons), Guy’s and St Thomas’ NHS Foundation Trust, London UK; Christopher Blazes, MD, Oregon Health Sciences University and Veterans Administration Medical Center, both in Portland, Oregon USA; Leslie Brooks, MD, Sunrise Community Health and North Colorado Health Alliance, Evans, Colorado USA; Jaden Brandt, Msc.Pharm, University of Manitoba College of Pharmacy, Winnipeg, Manitoba Canada; Cathal Cadogon, PhD, School of Pharmacy and Pharmaceutical Sciences, Trinity College Dublin, Ireland; Doryn Davis Chervin, DrPH, Chervin and Associates, Cherry Hill, New Jersey USA; David Crabtree, MD, QuitGenius and PlushCare, San Francisco, California USA; Tim MacDonald, MD, Griffith University and Currumbin Clinic, Currumbin, Queensland Australia; Darrin Mangiacarne, DO, MPH, DFASAM, FAOAAM, Banyan Treatment Centers,  Indianapolis, Indiana USA; Lori Mor, PharmD, Prisma Health Midlands, Family Medicine Residency Program, Florida, USA; Chinyere Ogbonna, MD, MPH, Kaiser Permanente, San Jose and Stanford Health, Stanford, both in California USA; Jocelyn Pederson; Arwen Podesta, MD, Tulane University School of Medicine, New Orleans, Louisiana USA; Erick Turner, MD, Department of Psychiatry, Oregon Health & Science University, Portland, Oregon USA; Jayne Violette, PhD, University of South Carolina Beaufort, Bluffton, South Carolina USA; and Steven Wright, MD [retired]. Bernard Sivernail served as moderator.
