# Supplementary material for: Long-term consequences of benzodiazepine-induced neurological dysfunction: A survey
Source: PLoS One. 2023 Jun 29;18(6):e0285584. doi: 10.1371/journal.pone.0285584 (PMC10309976; doi:10.1371/journal.pone.0285584)
Supplement: Attachment — Submitted filename: 3.24.23_Benzo #3 Rebuttal Letter Worksheet for Peer Review.docx [file pone.0285584.s003.docx]

| **Reviewer’s Comments** | **Authors’ Response** | **Action(s) Taken** |
| --- | --- | --- |
| *Editor* | | |
| Please ensure you meet PLOS One’s style guide | Yes | Done (minor editorial changes) |
| Competing Interest statement:  "I have read the journal's policy and the authors of this manuscript have the following competing interests: Alexis Ritvo is contracted as the medical director for the national non-profit the Alliance for Benzodiazepine Best Practices. Alexis Ritvo and D Foster are co-chairs for the Benzodiazepine Action Work Group with the Colorado Consortium for Prescription Drug Abuse Prevention. D Foster is also the founder and owner of Easing Anxiety. Christy Huff is a director with the Benzodiazepine Information Coalition. Bernie Silvernail ~~Sanders~~ is president of the Alliance for Benzodiazepine Best Practices. This does not alter our adherence to PLOS ONE policies on sharing data and material" | Please note changes | Highlighted |
| Please confirm that this does not alter your adherence to all PLOS ONE policies on sharing data and materials, by including the following statement: "This does not alter our adherence to  PLOS ONE policies on sharing data and materials.” (as detailed online in our guide for authors [http://journals.plos.org/plosone/s/competing-interests](https://nam12.safelinks.protection.outlook.com/?url=http%3A%2F%2Fjournals.plos.org%2Fplosone%2Fs%2Fcompeting-interests&data=05%7C01%7Cpeter.martin%40vumc.org%7C058772b130644357703508db27201572%7Cef57503014244ed8b83c12c533d879ab%7C0%7C0%7C638146790994196349%7CUnknown%7CTWFpbGZsb3d8eyJWIjoiMC4wLjAwMDAiLCJQIjoiV2luMzIiLCJBTiI6Ik1haWwiLCJXVCI6Mn0%3D%7C3000%7C%7C%7C&sdata=qZRFxWtbkvmeXbDE%2BD1tMmYEEYmU1nuDCVVe6dVGzV8%3D&reserved=0)).  If there are restrictions on sharing of data and/or materials, please state these. Please note that we cannot proceed with consideration of your article until this information has been declared. | [http://journals.plos.org/ plosone/s/competing-interests](http://journals.plos.org/%20plosone/s/competing-interests)).  The included link is on competing interests. Not sure how this applies to data sharing?  We acknowledge that we will share data | We acknowledge we will share data |
| We note that you have stated that you will provide repository information for your data at acceptance. Should your manuscript be accepted for publication, we will hold it until you provide the relevant accession numbers or DOIs necessary to access your data. If you wish to make changes to your Data Availability statement, please describe these changes in your cover letter and we will update your Data Availability statement to reflect the information you provide. | Yes, we intend on posting our dataset on the Alliance for Benzodiazepine Best Practices website in a protected area. | In progress… |
| Please include your full ethics statement in the ‘Methods’ section of your manuscript file. In your statement, please include the full name of the IRB or ethics committee who approved or waived your study, as well as whether or not you obtained informed written or verbal consent. If consent was waived for your study, please include this information in your statement as well. | Yes, a full ethics statement was already included in the initial draft. | Done |
| Please include captions for your Supporting Information files at the end of your manuscript, and update any in-text citations to match accordingly. Please see our Supporting Information guidelines for more information: [http://journals.plos.org/plosone/s/supporting-information](https://nam12.safelinks.protection.outlook.com/?url=http%3A%2F%2Fjournals.plos.org%2Fplosone%2Fs%2Fsupporting-information&data=05%7C01%7Cpeter.martin%40vumc.org%7C058772b130644357703508db27201572%7Cef57503014244ed8b83c12c533d879ab%7C0%7C0%7C638146790994196349%7CUnknown%7CTWFpbGZsb3d8eyJWIjoiMC4wLjAwMDAiLCJQIjoiV2luMzIiLCJBTiI6Ik1haWwiLCJXVCI6Mn0%3D%7C3000%7C%7C%7C&sdata=EJx5omrm6%2BKqyEo1YliUuncKgyUVBfA74InZPrJMjF0%3D&reserved=0). | Yes | Done |
| We note that you have referenced (unpublished on page 13) which has currently not yet been accepted for publication. Please remove this from your References and amend this to state in the body of your manuscript: (ie “Bewick et al. [Unpublished]”) as detailed online in our guide for authors  <http://journals.plos.org/plosone/s/submission-guidelines#loc-reference-style> | This report is available online and is cited as such. | Done. |
| *Reviewer #1* | | |
| It was my pleasure to review the manuscript Long-term Consequences of Benzodiazepine-Induced Neurological Dysfunction: A Survey. The article revolves around a sound and painstaking analysis of 1,207 benzodiazepine users from benzodiazepine support groups and health/wellness sites. The article is quite informative and cogently enunciated in its most relevant findings, which are ultimately a worthy research contribution likely to appeal to a rather broad readership of mental health professionals and addiction specialists. Shedding a light on the underlying factors determining or contributing to neurological repercussions from BDZ use is essential for forensic medicine and public health as well, given the far-reaching implications thereof. | Thank you. | None |
| In that regard, I feel that the article does not go far enough, and does not fully succeed in making the most out of its data analysis. More depth needs to be added to the Discussion, also mentioning "substitute" BDZ substances and the threat they pose in terms of detection and control, in addition to the psychiatric implications. | Agreed, will add, but we wish to note that our survey did not specifically address “designer benzos” although it is possible some of our respondents had experience with them. | Added |
| The reference pool should be enhanced, consider the following:  PMID: 32144953. PMID: 29543325 PMID: 31799633. PMID: 36041417. | We appreciate this and have reviewed these materials. | Added text and references |
| The article is clear and straightforward overall, and the tables are meaningful and well conceived. Making it more comprehensive will add to its relevance and improve balance and development. | Thank you. We are agreeable with this addition providing it is noted that our survey did not specifically ask about designer benzos. Further, based on the survey responses, most respondents likely took prescribed pharmaceutical drugs under the supervision of their clinical team. | None |
| *Reviewer #2* | | |
| I have read and mostly appreciated your article titled Long-term *Consequences of Benzodiazepine-Induced Neurological Dysfunction: A Survey*, in which the distinctive features and complex dynamics at the heart of BIND have been expounded upon quite effectively and in a scientifically sound fashion. | Thank you | None |
| The article has qualities and strengths which make it a praiseworthy scientific research contribution. It has considerable elements of novelty, relevance and thoroughness as far as its stated objective is. The methodology is sound, as far as I could determine. | Thank you | None |
| The one area in which the article falls short is the limited scope in terms of mentioning and elaborating on factors such as screening and detection and policies and measures aimed at mitigating the spread of BDZs with an eye on designer drugs, i.e. replacements to BDZs and other substances of abuse. | Yes, this point was raised with Reviewer #1 as well and will be addressed. | We added a section |
| Briefly addressing such elements of discussion would certainly contribute to making the article more comprehensive and well-rounded, which would be advisable in light of the uniquely consequential issues arising from BDZs abuse. It is also worth mentioning the impact of the COVID-19 pandemic on abuse dynamics overall. Too many sources are older than five years. | The impact of COVID-19 on abuse dynamics is not well studied, particularly as it related to benzodiazepines. This is an important and intriguing topic but one that seems outside the scope of our article. However, in light of the relevance and value of this topic and its timeliness, we are comfortable mentioning it, but our survey was conducted pre-pandemic and we feel that introducing too much peri-pandemic information would be misleading. | We added a sentence on this, but it is important to note our study was completed prior to COVID-19. |
| The following sources ought to be drawn upon and cited as well:  Zaami S, Graziano S, Tittarelli R, Beck R, Marinelli E. BDZs, Designer BDZs and Z-drugs: Pharmacology and Misuse Insights. Curr Pharm Des. 2022;28(15):1221-1229. doi: 10.2174/1381612827666210917145636.  Moosmann B, Auwärter V. Designer Benzodiazepines: Another Class of New Psychoactive Substances. Handb Exp Pharmacol. 2018;252:383-410. doi: 10.1007/164_2018_154.  Lo Faro AF, Venanzi B, Pilli G, Ripani U, Basile G, Pichini S, Busardò FP. Ultra-high-performance liquid chromatography-tandem mass spectrometry assay for quantifying THC, CBD and their metabolites in hair. Application to patients treated with medical cannabis. J Pharm Biomed Anal. 2022 Aug 5;217:114841. doi: 10.1016/j.jpba.2022.114841.  Negro F, Di Trana A, Marinelli S. The effects of the COVID-19 pandemic on the use of the performance-enhancing drugs. Acta Biomed. 2022 Jan 19;92(6):e2021401. doi: 10.23750/abm.v92i6.12377.  Mannocchi G, Di Trana A, Tini A, Zaami S, Gottardi M, Pichini S, Busardò FP. Development and validation of fast UHPLC-MS/MS screening method for 87 NPS and 32 other drugs of abuse in hair and nails: application to real cases. Anal Bioanal Chem. 2020 Aug;412(21):5125-5145. doi: 10.1007/s00216-020-02462-6.  Walton SE, Krotulski AJ, Logan BK. A Forward-Thinking Approach to Addressing the New Synthetic Opioid 2-Benzylbenzimidazole Nitazene Analogs by Liquid Chromatography-Tandem Quadrupole Mass Spectrometry (LC-QQQ-MS). J Anal Toxicol. 2022 Mar 21;46(3):221-231. doi: 10.1093/jat/bkab117.  Napoletano S, Basile G, Lo Faro AF, Negro F. New Psychoactive Substances and receding COVID-19 pandemic: really going back to "normal"? Acta Biomed. 2022 May 11;93(2):e2022186. doi: 10.23750/abm.v93i2.13008. | These articles are of great interest but appear to divert us from our primary theme. There has been no research we could find specifically addressing changes in benzodiazepine use during the pandemic. The pandemic’s effects on use patterns of cannabinoids, performance-enhancing drugs, nitazenes, etc. are not comparable to benzodiazepines. Most of our respondents were taking prescribed drugs under the supervision of a trusted physician (this would likely not be the case with some of these references). Since our survey did not ask about the use of these agents and since it was conducted pre-pandemic, the introduction of how the pandemic impacted, say, the use of performance-enhancing drugs, seems very far afield. Neither the timing nor the drugs are similar.  We are agreeable to mention designer benzodiazepines, as this is an important topic, but to devote more references to this topic than we used for on-target topics seems to skew our findings. Our goal was not to investigate pandemic-related drug behaviors---the survey concluded prior to the outbreak of COVID-19. We did not research or ask about designer benzodiazepines, much less nitazenes or other agents. | Added a paragraph and references. |
| 6. PLOS authors have the option to publish the peer review history of their article ([what does this mean?](https://nam12.safelinks.protection.outlook.com/?url=https%3A%2F%2Fjournals.plos.org%2Fplosone%2Fs%2Feditorial-and-peer-review-process%23loc-peer-review-history&data=05%7C01%7Cpeter.martin%40vumc.org%7C058772b130644357703508db27201572%7Cef57503014244ed8b83c12c533d879ab%7C0%7C0%7C638146790994196349%7CUnknown%7CTWFpbGZsb3d8eyJWIjoiMC4wLjAwMDAiLCJQIjoiV2luMzIiLCJBTiI6Ik1haWwiLCJXVCI6Mn0%3D%7C3000%7C%7C%7C&sdata=fN6rTrPPmgeZz%2BXOstE1uVjgoBzHKWMxY16BmTFFMKc%3D&reserved=0)). If published, this will include your full peer review and any attached files.   If you choose “no”, your identity will remain anonymous but your review may still be made public.   **Do you want your identity to be public for this peer review?** For information about this choice, including consent withdrawal, please see our [Privacy Policy](https://nam12.safelinks.protection.outlook.com/?url=https%3A%2F%2Fwww.plos.org%2Fprivacy-policy&data=05%7C01%7Cpeter.martin%40vumc.org%7C058772b130644357703508db27201572%7Cef57503014244ed8b83c12c533d879ab%7C0%7C0%7C638146790994196349%7CUnknown%7CTWFpbGZsb3d8eyJWIjoiMC4wLjAwMDAiLCJQIjoiV2luMzIiLCJBTiI6Ik1haWwiLCJXVCI6Mn0%3D%7C3000%7C%7C%7C&sdata=8h%2F5gUSEXPqhNND4Ve6dhrmP3sGevTZdTp%2FRT6z8IGQ%3D&reserved=0).  Reviewer #1: No  Reviewer #2: No | Noted. | None |
